# Development of a rapid and efficient protoplast isolation and transfection method for chickpea (*Cicer arietinum*)

**DOI:** 10.1016/j.mex.2020.101025

**Published:** 2020-08-08

**Authors:** Ninghui Cheng, Paul A. Nakata

**Affiliations:** Department of Pediatrics, Baylor College of Medicine, USDA/ARS Children's Nutrition Research Center, Houston, TX, United States

**Keywords:** Protoplast, Transfection, Chickpea, Transient expression, Subcellular localization

## Abstract

Chickpea (*Cicer arietinum* L.) is the second most important grain legume worldwide. Recent advances in the sequencing of the chickpea genome has provided a new and valuable resource to aid efforts in gene discovery and crop trait improvement. Technical difficulties in stable chickpea transgenics and the lack of a transient expression system for rapid analysis of gene expression and function; however, has limited the usefulness of this genomic resource. As a step toward alleviating this limitation, we report here the development of a simple and efficient transient gene expression protocol. Using leaves from chickpea seedlings, we have established a procedure that enables the generation of large quantities of vital chickpea protoplasts within only a few hours. In addition, we have optimized a PEG-calcium-mediated transfection method to efficiently deliver exogenous DNA into the chickpea protoplast. The current study is the first to present a detailed step-by-step procedures for protoplast isolation, evaluation, transfection, and application in chickpea. In addition, we optimize the transfection efficiency which has not been previously reported. Our protoplast transfection approach provides a platform that will allow rapid high-throughput screening and systematic characterization of gene expression and function. Knowledge gained through such studies will benefit current efforts to improve chickpea production and quality.

• Modified enzymatic digestion solution for higher yield and viability.
• Optimize transfection of chickpea protoplasts.

## Graphical abstract

**Figure:**
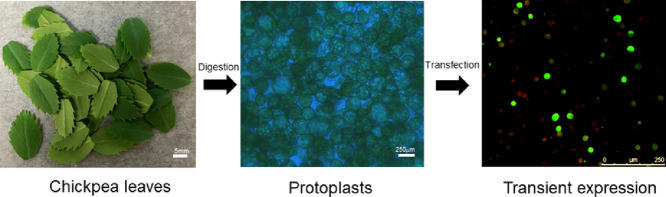

Specifications table

**Table:** 

Subject Area:	Biochemistry, Genetics and Molecular Biology
More specific subject area:	Plant Molecular Biology
Method name:	Isolation and transfection of Chickpea protoplast
Name and reference of original method:	Shu Y, Huang L, Chen M, Tao Y, Wang Z, Ma H. Establishment and optimization of systems for protoplasts isolation of soybean and chickpea that used in subcellular location. Shengwu Gongcheng Xuebao/Chinese Journal of Biotechnology. 2017;33:976–85. doi: 10.13345/j.cjb.170086.
Resource availability:	*NA*

## Method details

### Reagents

• Cellulase Onozuka R-10 (PhytoTechnology Laboratories, Shawnee Mission, KS, USA).
• Macerozyme R-10 (A mixture of Pectinase, Cellulase and Hemicellulase; PhytoTechnology Laboratories).
• 2-(N-Morpholino)ethanesulfonic acid hydrate (MES, Sigma-Aldrich, St. Louis, MO, USA, Cat. no. M8250).
• D-Mannitol (Sigma-Aldrich, Cat. no. M1902).
• Polyethylene glycol 4000 (PEG, Sigma-Aldrich,Cat. no. 95904).
• Potassium chloride (KCl, Sigma-Aldrich, Cat. no. P3911).
• Calcium chloride dihydrate (CaCl_2_ 2H_2_O, Sigma-Aldrich,Cat. no. C7902).
• Sodium chloride (NaCl, EMD, Merck, Kenilworth, N.J., USA).
• Magnesium chloride hexa-hydrate (MgCl_2_.6 H_2_O, VWR, Missouri, TX, USA, BDH-0244,).
• DL-Dithiothreitol (DTT, Sigma-Aldrich, Cat no. D0632).
• Propidium iodide (PI, Sigma-Aldrich, Cat. no P4170).
• Fluorescein diacetate (FDA, Sigma-Aldrich, Cat. no. F7378).
• Acetone (Sigma-Aldrich, Cat. no. 650501).
• Bovine serum albumin (BSA, EMD, Merck).
• Calf serum (Gibco, Thermo Fisher Scientific, Waltham, MA USA).
• Phosphate buffered saline (PBS, Gibco, Thermo Fisher Scientific).
• QIAprep Spin Miniprep Kit (QIAGEN, Germantown, MD, Cat. no. 27104).

### Reagents setup

• **Enzyme solution (Digestion solution):** Prepare 5 mM MES (pH 6.0) containing 1% (w/v) cellulase R-10, 0.8% (w/v) macerozyme R-10, 0.55 M d-Mannitol, 0.2 mM CaCl_2_, 0.5 mM DDT and 0.1% (w/v) BSA. Warm the solution at 55 °C for 10 min to inactivate DNase and proteases, and then cool it to room temperature before use. The enzymatic digestion solution should be freshly prepared.
• **W5 solution:** 2 mM MES (pH 6.0), 154 mM NaCl, 125 mM CaCl_2_, and 5 mM KCl. The prepared W5 solution can be stored at 4 °C.
• **MMg solution:** 4 mM MES (pH 6.0), 0.4 M d-Mannitol, and 15 mM MgCl_2_. The prepared MMg solution can be stored at room temperature.
• **WI solution:** 4 mM MES (pH 6.0), 0.5 M d-Mannitol, and 20 mM KCl. The prepared WI solution can be stored at room temperature.
• **PEG–calcium transfection solution:** 20%, 30%, 40%, and 50% (w/v) PEG4000, 0.2 M d-Mannitol, and 100 mM CaCl_2_. PEG solution should be always freshly prepared.
• **Plate coating solution:** 5% (v/v) sterile calf serum in 1x PBS. The solution should be stored at 4 °C.
• **PI solution:** The stock solution is 1 mg ml^−1^ in H_2_O and should be stored at 4 °C. The final concentration is 5 µg ml^−1^.
• **FDA solution:** The stock solution is 25 mg ml^−1^ in acetone and should be protected from light and stored at −20 °C. The working solution is 50 µg ml^−1^.

### Equipment

• Single edge industrial razor blades (VWR, Missouri, TX, USA, Cat no. 55411-050).
• Fluorescence microscope (Olympus CKX41, Olympus, Center Valley, PA, USA).
• Confocal laser scanning microscope (Leica SM8, Leica, Wetzlar, Germany).
• Bench Centrifuge with swinging-bucket rotor (Sovrall Legend RT, Kendro Laboratory Products, Newtown, CT, USA).
• Centrifuge 5415 R (Eppendorf, Hamburg, Germany).
• Orbital Incubator shaker for bacterial growth (GyroMax 737, Amerex Instruments Inc, Concord, CA, USA).
• Orbital shaker for protoplast digestion (New Brunswick Scientific, Edison, NJ).
• pH meter (Accumet AE150, Fisher Scientific).
• 14-ml polystyrene round-bottomed tube (Becton, Dickinson Labware, NJ, USA).
• Cell Strainer, (70 µm nylon mesh, Fisher Scientific, Cat. no. 22363548,).
• 12-well Falcon Plates (Cat. no. 351143, Corning Incorporated, Corning, NY. USA).
• Petri dishes (60 × 15 mm, cat. no. 430196, Corning Incorporated).
• Glass bottom dish (35 mm dish with 14 mm bottom well, In Vitro Scientific, Cat. no. D35-14-1-N).
• Bright-Line Hemocytometer (Sigma-Aldrich, Cat. no. Z359629).
• Microscope cover glass (VWR, Cat. no. 48366067).
• Vacuum desiccator (Bel-Art Products, Pequannock, NJ, USA, Cat. no. F42027).
• Vacuum pump (Welch vacuum, Thomas Industries Inc., Skokie, IL, USA).

### Protocol

**Fig. 1:**
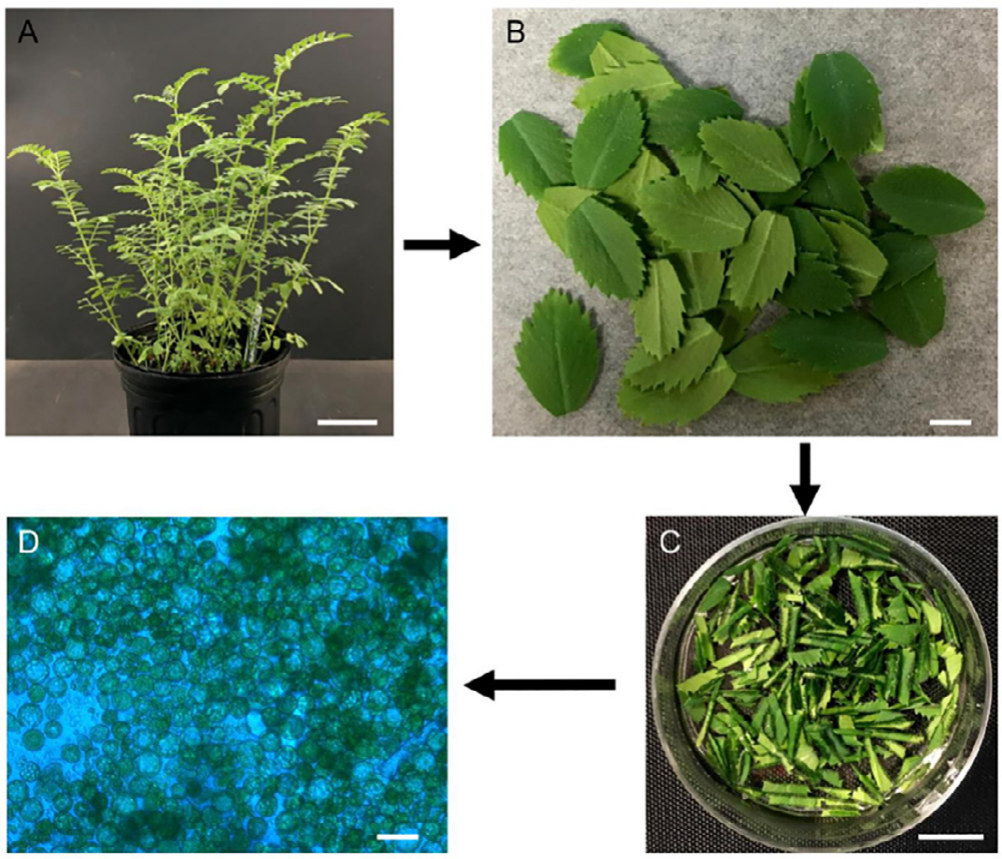
A flowchart depicting the chickpea protoplast isolation procedure. (**A**) Three week old chickpea plants. Scale bar=5 cm. (**B**) Harvested chickpea leaves. Scale bar=5 mm. (**C**) Enzymatic digestion of chickpea leaves. Scale bar=1 cm. (**D**) Isolated chickpea protoplasts. Scale bar=25 µm.

1. **Growth of the chickpea plants**

(1) 4–6 chickpea seeds (CDC Frontier) were sown on commercial soil (Pro-Line, growing mix, C/20, Jolly Gardener, Oldcastle Lawn & Garden, Inc., ME, USA) in 16 cm pots and grown in the greenhouse under long-day growing condition (16/8 h photoperiod; light range from 686 to1400 µmolm^−2^s^−1^) at 26/18 °C for 2–4 weeks (Fig. 1A).

**Critical:** Healthy chickpea plants grown without biotic or abiotic stresses are important for protoplast isolation and high transfection efficiency.

2. **Preparation of plasmid DNA**
(1) *E. coli* (TOP10) transformed with pRTL2-GFP [1], pRTL2-GFP-CXIP4 [1], or pRTL2-AtGrxS16-GFP [2] constructs were grown in 20 mL Luria–Bertani (LB) liquid medium supplemented with 100 µg mL^−1^ ampicillin. The cultures were grown in a 100 mL flask with shaking at 160 rpm at 37 °C for 14 h to an OD=2.0.
(2) Plasmid DNA was extracted and purified from *E. coli* using the QIAprep Spin Miniprep Kit following the manufacturer's procedure. The plasmid DNA concentration was adjusted to 0.5 µg µL^−1^ for transfection.

**Critical:** High concentrations of purified supercoil plasmid DNA are crucial for protoplast transfection. Using commercial plasmid DNA purification kit, like QIAGEN kits, is recommended.

3. **Protoplast isolation from chickpea leaves**
(1) 0.5 g of fully expanded leaves was harvested from 2 to 3 week old chickpea seedlings (Fig. 1B).

**Critical:** Using leaves harvested from the middle leaf position of a young chickpea plant is key to successful chickpea protoplast isolation and transfection.

(2) Leaves (entire leaflet) were cut into 0.5–1.0 mm strips using a razor blade and transferred to a petri dish containing 5 mL of freshly prepared digestion solution (Fig. 1C).
(3) The petri dish containing the leaf strips was placed in the vacuum desiccator, connected to a vacuum pump, and vacuumed infiltrated for 20–30 min at room temperature.

**Critical:** Infiltration of leaf tissues is critical for the quick release and yield of mesophyll protoplasts.

(4) Leaf strips were incubated in the digested solution at room temperature with gentle shaking (50 rpm) under low light for 2–3 h.

**Critical:** Monitor the digestion process using a light microscope (Fig. 1D). The released protoplasts look spherical, while the undigested cells appear more irregular in shape. Avoid over digestion and long incubation times.

(5) Leaf digestion was terminated by first mixing 5 mL of W5 solution into the leaf digestion mixture. Next, this entire mixture was gently passed through a clean 70 µm nylon mesh. This filtering process allowed the removal of the undigested leaf tissues and debris while the released protoplasts passed through the mesh.
(6) The digested protoplast were transferred to 14-mL round-bottom tube using a wide-end tip pipet and the tube centrifuged in a swinging-bucket rotor at 100 × *g* for 4 min at room temperature.

**Critical:** Set centrifuge acceleration and deceleration rates at the lowest setting to minimize protoplast damage during the centrifugation. Using the round-bottom tube and swinging-bucket rotors are highly recommended.

(7) The supernatant was slowly removed without disturbing the protoplast pellet. Then the protoplast pellet was gently resuspended using 10 mL of cold W5 solution. The resuspended protoplast mixture was kept on ice for 30 min.

**Critical:** During protoplast resuspension slowly add 1 mL of W5 solution first along the side of tube and rotate the tube to release the protoplasts from the pellets before adding the additional 9 mL of W5 solution.

(8) Protoplast yields were determined using a hemocytometer and a light microscope.
(9) Protoplasts viability was assessed by staining with FDA and PI fluorescent dyes. 5 µL of PI stock solution and 4 µL of FDA stock solution were separately mixed into 1 mL of 0.55 M mannitol. 10 µL of each dye solution was then added to 20 µL of the protoplast mixture, and visualized using confocal microscopy. Living protoplasts stained with FDA showed a green fluorescence while dead protoplasts stained with PI displayed a red fluorescence (nuclei).
(10) After incubating the protoplast mixture on ice for 30 min the mixture was centrifuged at 100 × *g* for 4 min at room temperature and the W5 solution removed using a 1 mL pipette without disturbing the protoplast pellets.
(11) The protoplast pellet then was gently resuspended in MMg solution to a concentration of 1 × 10^6^ protoplast mL^−1^. Protoplast viability was determined as described in Step 9.

**Critical:** Chickpea mesophyll protoplasts appear to be sensitive to MMg solution. Thus, it is highly recommended to minimize exposure to this solution by proceeding to PEG-calcium mediated transfection as soon as possible.

4. **PEG-calcium mediated protoplast transfection**
(1) 200 µL of protoplasts (2 × 10^5^ protoplasts at 1 × 10^6^ mL^−1^) were aliquoted into 14 mL round-bottom tubes. One aliquot was set aside to serve as negative control.
(2) 10 or 20 µL of plasmid (5 or 10 µg) was gently mixed into each aliquot of protoplasts.

**Critical:** The size of plasmid DNA construct impacts the transfection efficiency. Smaller size constructs have a better transfection efficiency than larger size constructs. Thus, the amount of plasmid DNA used should be optimized empirically.

(3) 210 or 220 µL of freshly prepared PEG-Calcium solution was slowly added down the side of the tube and then gently rotated to mix well.

**Critical:** The concentration of PEG-calcium solution should be optimized empirically.

(4) The transfection reaction was incubated in the dark at room temperature for 15 min.

**Critical:** The incubation time should be optimized empirically.

(5) After incubation, 950 µL of W5 solution was slowly added to each tube, and then gently mixed well.
(6) The tubes were centrifuged at 100 × *g* for 4 min at room temperature. After centrifugation the supernatant then was carefully removed using a pipette.
(7) The transfected protoplast pellets then were gently resuspended in 1 mL of WI solution.
5. **Protoplast incubation and harvest**
(1) One mL of 5% (v/v) sterile calf serum was added into each well of a 12-well tissue culture plate to coat the surface and prevent the protoplasts from sticking to the plate surface. After a 1 min incubation the coating solution was aspirated.
(2) The protoplasts from each tube was transferred to each well of the culture plate. The plate was covered and the lid sealed with the surgical tape.
(3) The protoplasts were incubated at room temperature for 12–18 h in the dark.

**Critical:** Chickpea mesophyll protoplasts in WI solution can live for at least 24 h. In general, longer incubation times (16–18 h) usually yields stronger fluorescent signals.

(4) The protoplasts then were collected by transferring the protoplast solution to 14 mL round-bottom tubes and centrifuging at 100 × *g* for 4 min at room temperature.
(5) Most of the supernatant was removed and the protoplasts resuspended in the residual supernatant harvested using a wide-end pipette.
6. **Imaging of fluorescent signals**
(1) 50 µL of transfected protoplasts were transferred to a glass bottom dish and covered with a glass coverslip.
(2) The fluorescence signals were detected at 510 nm (excitation at 488 nm) for GFP or FDA, 582 nm (excitation at 543 nm) for PI, and 660 nm (excitation at 633 nm) for chlorophyll using Leica SM8 confocal laser-scanning microscope.

## Method validation

Reliable transient expression systems have proven invaluable in the study of gene expression, gene function, protein localization, and protein–protein interactions. Such expression systems are especially important in plants, such as chickpea, that lack a simple and efficient method of stable plant transformation. Thus, we report here the establishment of a fast and reliable protocol for transient gene expression in chickpea. With the recent advances in chickpea genome sequencing [3,4] such a protocol is needed to help expedite efforts in gene discovery and crop trait improvements.

### Protoplast isolation

**Fig. 2:**
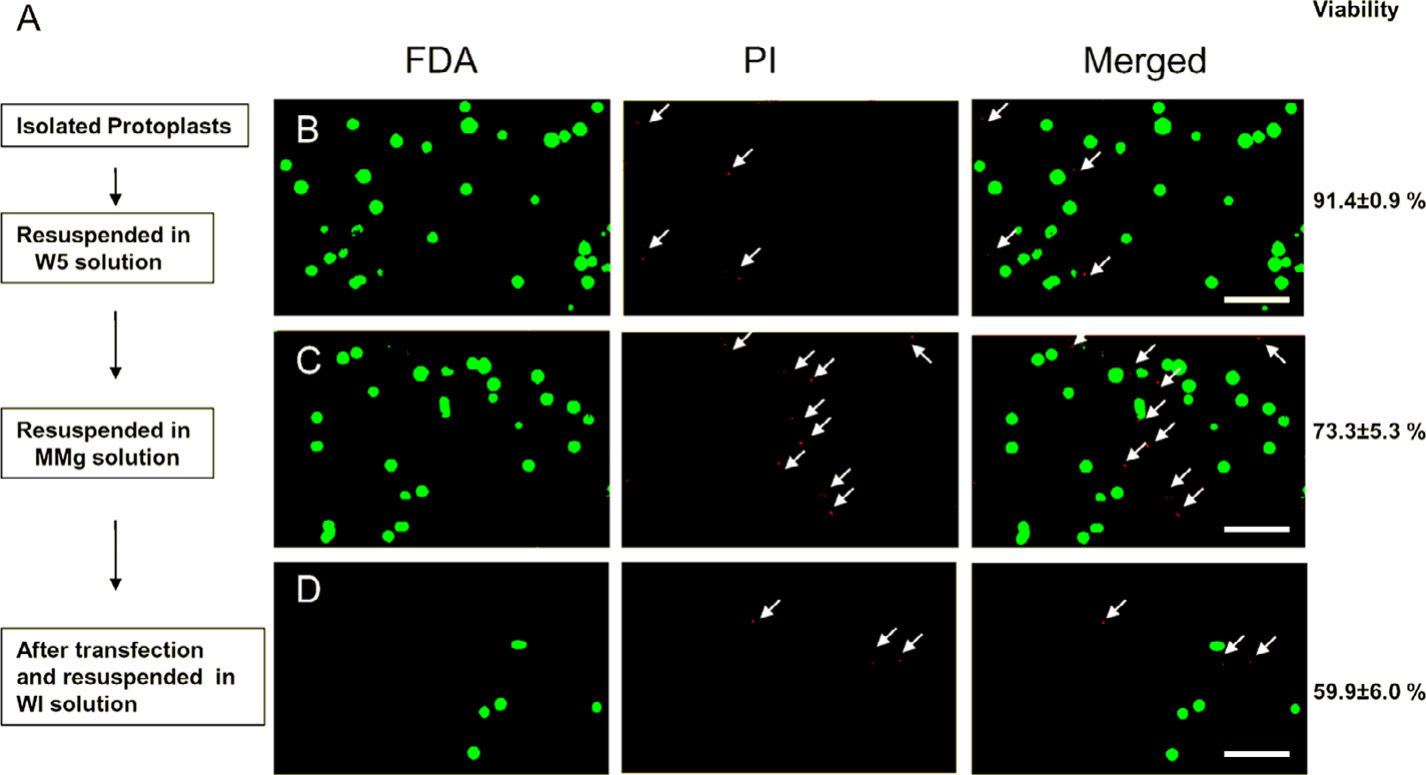
Protoplast viability during preparation and transfection. (**A**) Major steps for protoplast transfection. (B-D) Protoplast staining with FDA and PI. Living protoplasts stained green (FDA) while dead protoplasts were stained red (PI, arrows). Scale bar=250 µm. (For interpretation of the references to color in this figure legend, the reader is referred to the web version of this article.)

Young fully expanded leaves (Fig. 1A and B) were selected as source tissue for chickpea protoplast preparations. Leaf pieces were digested with a mixture of cell wall degrading enzymes resulting in the release of large quantities of mesophyll protoplasts (1.36±0.2 × 10^7^ protoplasts) from just a few hundred mg of source tissue (Fig. 1D and Fig. 2). Protoplasts isolated from more mature leaves (more than 4 weeks) is possible, however, use of this older source tissue resulted in lower protoplast yields compared to preparations conducted utilizing younger source leaves (data not shown).

### Protoplast viability

Viability of the isolated chickpea protoplasts was monitored at different steps in the transfection process (Fig. 2A). The two dyes, FDA and PI, were utilized to distinguish between viable and nonviable protoplasts, respectively [5]. Viability of the protoplasts released from the digestion of the source leaves was determined to be 91.4 ± 0.9% after washing and resuspension in W5 solution (Fig. 2B). This finding is similar to the percentages reported in protoplast preparation conducted in other legumes [6], [7], [8], [9]. In preparation for transfection the protoplast were next re-suspended in MMg solution. Staining of this cell population revealed a 73% viable protoplast population (Fig. 2C). Upon completion of the transfection procedure vital staining indicated that about 60% of the protoplasts remained intact and viable (Fig. 2D). This observed decline in protoplast viability at each step in the transfection process has been similarly reported [5].

### Protoplast transfection

**Fig. 3:**
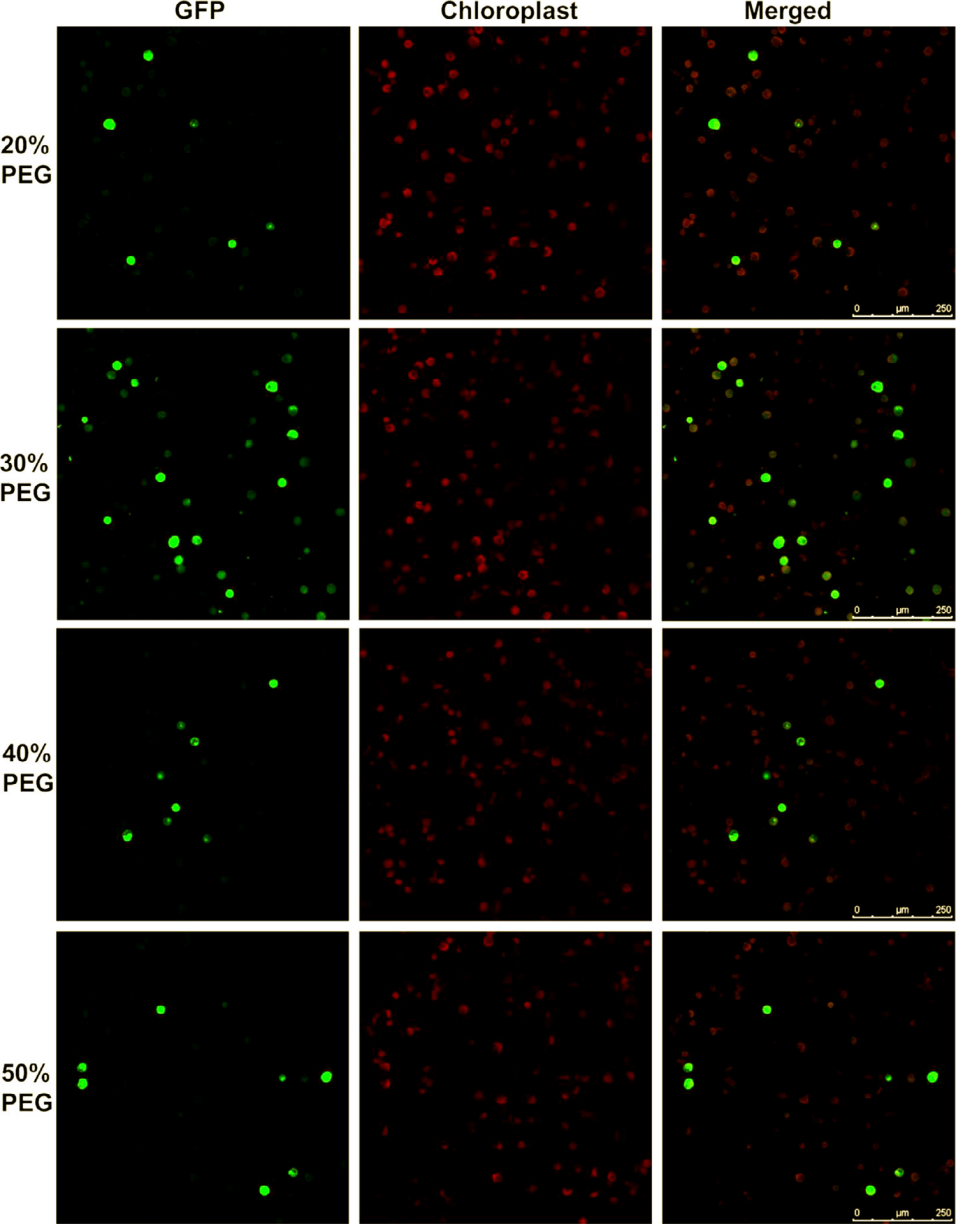
Efficiency of chickpea protoplast transfection. Chickpea protoplasts were transfected with 10 µg of pRTL2-GFP using different concentrations (20%, 30%, 40%, and 50%) of PEG. Confocal images visualizing GFP fluorescence (left), chloroplasts auto-fluorescence (middle) and the two fluorescent signals merged (right). The transfection efficiency was assessed by the number of GFP fluorescent protoplasts. Fluorescent signals of GFP fusion proteins were imaged 16 h after transfection. Scale bars=250 µm.

It has been reported that the concentration of PEG used in protoplast transfection is a critical factor in determining transfection efficiency [10], [11], [12], [13]. Thus, using a GFP expression construct driven by the 35S CAMV promoter (pRTL2-GFP) [1], we assessed the efficiency of chickpea transfection using different concentrations of PEG (from 20% to 50%). Previous reports demonstrated that greater than a 50% transfection rate was achieved in soybean and Medicago protoplasts [8,9] using 20% PEG while in other legumes such as common bean (*Phaseolus vulgaris*), the use of a 40% PEG solution was found to result in the highest transfection efficiency [6]. Optimization of chickpea protoplast transfection; however, has yet to be reported. As shown in Fig. 3, we determined that the use of a 30% PEG solution resulted in the highest transfection efficiency in chickpea with more than a 50% transfection efficiency. In addition, we determined that the amount of plasmid DNA used for protoplast transfection also influenced the transfection efficiency. The use of 10 µg of plasmid DNA (Fig. 3) resulted in higher transfection efficiency than 5 µg of plasmid DNA (S1 Fig). This finding of higher transfection efficiency with larger amounts of plasmid DNA is consistent with the findings reported in a study using soybean protoplasts [9].

### Subcellular protein localization

**Fig. 4:**
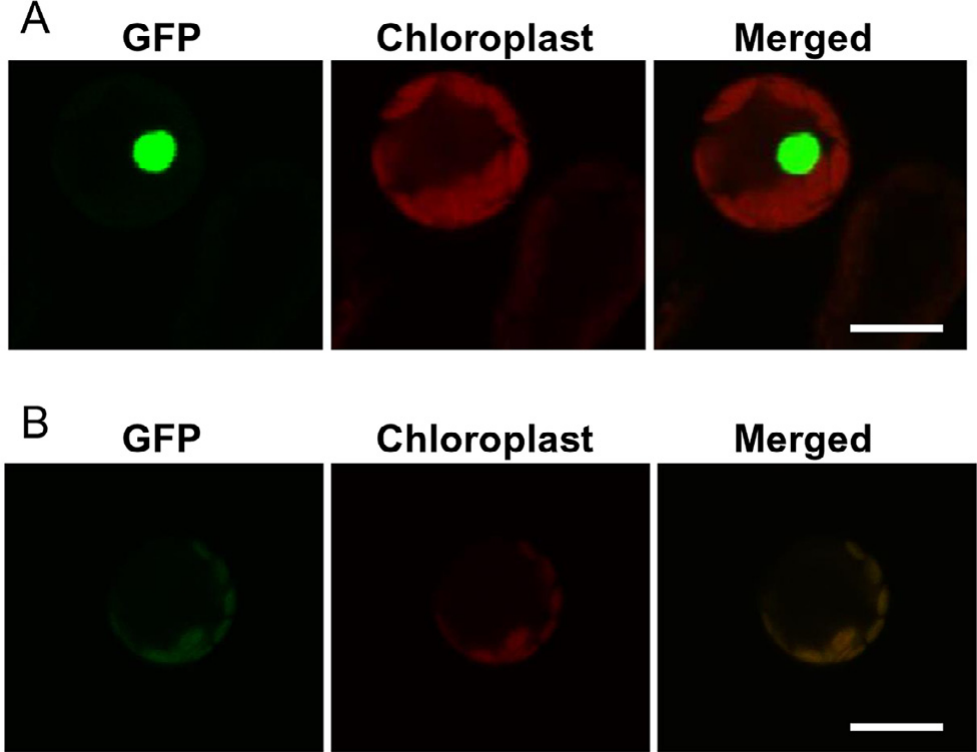
Subcellular localization of protein markers. (**A**) Nuclear localization of CXIP4-GFP fusion protein in chickpea protoplasts. (**B**) Chloroplast localization of GrxS16-GFP fusion in chickpea protoplasts. Fluorescent signals of GFP fusion proteins were imaged 16 h after transfection. Scale bars=10 µm.

To determine whether the prepared chickpea protoplasts were suitable for subcellular localization studies, nuclear and plastid target marker proteins were expressed in the prepared protoplasts. As shown in Fig. 4, transfection of the chickpea protoplasts with a construct encoding a GFP fusion to CXIP4 was successfully expressed and targeted to the nucleus of the chickpea cell. CXIP4 has been shown to contain a nuclear localization signal [1,2] and this gene is conserved in different plants (S2 Fig). Transfection of a construct encoding a GFP fusion to *GrxS16* resulted in a marker protein localized to the chloroplast within the chickpea cell. GrxS16 proteins from different plants all contain a plastid targeting transit peptide [1,2] and are also conserved among plants (S2 Fig). Although heterologous expression systems have proven useful in a number of protein localization studies there have been instances of mislocalization and therefore misinformation [14], [15], [16]. Thus, use of homologous systems are preferred whenever possible. Overall, the findings from this study demonstrate that this transient gene expression protocol is suitable for conducting protein localization studies in chickpea.

## Conclusions

Transient expression systems have proven useful in rapid high-throughput screening and systematic characterization of genes and proteins in many plants. Such transient expression systems are especially important in plants where stable transformation protocols are time consuming and/or inefficient. It is our hope that the transient chickpea expression system presented in this report will be employed as a tool to help harness the increasing amounts of available genomic data and will be applied toward efforts to expedite gene discovery and crop trait improvements in this important legume.

## CRediT authorship contribution statement

**Ninghui Cheng:** Methodology, Investigation, Visualization, Writing - original draft. **Paul A. Nakata:** Conceptualization, Methodology, Writing - review & editing, Supervision, Resources.

## Declaration of Competing Interest

The authors declare that they have no known competing financial interests or personal relationships that could have appeared to influence the work reported in this paper.

## Appendix Supplementary materials

Image, application 1
