# Supplementary material for: Development of a rapid and efficient protoplast isolation and transfection method for chickpea (Cicer arietinum)
Source: MethodsX. 2020 Aug 8;7:101025. doi: 10.1016/j.mex.2020.101025 (PMC7452273; doi:10.1016/j.mex.2020.101025)
Supplement: Supplementary file 1 [file mmc1.docx]

**Supplementary material *and/or* Additional information:**

**Additional Information**

Chickpea (*Cicer arietinum* L.) is a cool-season pulse crop and the second most widely grown legume crop worldwide [17, 18]. Chickpea serves as a fundamental source of dietary fiber, protein, carbohydrates, vitamins and minerals for millions of people especially in developing countries [19-21]. In addition, chickpea crops can help in soil fertility management through symbiotic nitrogen fixation [22]. This is significant since the cultivation of chickpea often occurs in parts of the world that consist of semi-arid environments and soils of poor agricultural quality. Poor environmental conditions combined with the susceptibility of chickpea to drought and pathogens have limited yields below their theoretical potential [3]. Genetic improvements in plant productivity have been hampered by the limited introduction of genetically diverse germplasms for use by breeders and by the limited molecular-genetic resources available for use by plant biologists.

Recently, genome sequencing efforts have provided a new and essential molecular-genetic resource for chickpea crop trait improvement. These sequencing efforts revealed a chickpea genome size of about 738 Mb encoding an estimated 28,269 genes [3]. In addition, resequencing of 429 chickpea accessions from 45 countries has provided new insights into genome diversity, domestication, and agronomic traits [4]. Such advancements in chickpea genomics has provided a valuable resource to aid our efforts to identify and characterize genes encoding traits of agronomic importance [23, 24]. Efforts to fully harness this newly available genetic resource; however, have been impeded by the difficulties associated with the time consuming process of stable chickpea transformation and the lack of an efficient transient gene expression system.

Compared with stable gene expression, transient gene expression systems provide a simple, rapid, and efficient alternative [10, 25]. The use of plant protoplasts for transient gene expression is a proven technique for defining promoter regulation, gene expression, subcellular protein localization, protein-protein interactions, and cellular signaling pathways [25-27]. The use of transient expression systems have been further expanded to include genetic screens for gene silencing and genome-editing events [28-35]. Recently, efficient transient gene expression systems using protoplasts have been established in a number of crop species such as rice [16], tomato [36], soybean [7, 9], common bean [6], *Lotus* and *Medicago* [8]. A highly efficient transient transfection system for chickpea; however, has yet to be established.

In this study, we report the establishment of a simple and efficient transient gene expression system for chickpea. This system yields large quantities of vital protoplasts within only a few hours and has been optimized to deliver DNA into chickpea protoplasts with high efficiency. Overall, this transient chickpea gene expression system provides a convenient and versatile platform allowing for molecular, cellular, and functional studies to be quickly conducted in chickpea.

**References**

[17] K.K. Sharma, P. Bhatnagar-Mathur, B. Jayanand. Chickpea (Cicer arietinum L.). In: K Wang, editor. Agrobacterium Protocols. Totowa, NJ: Humana Press; 2006. p. 313-24.

[18] M. Asif, L.W. Rooney, R. Ali, M.N. Riaz, Application and Opportunities of Pulses in Food System: A Review, Crit Rev Food Sci Nutr 53 (11) (2013) 1168-79, doi: 10.1080/10408398.2011.574804.

[19] A.K. Jukanti, P.M. Gaur, C.L.L. Gowda, R.N. Chibbar, Nutritional quality and health benefits of chickpea (Cicer arietinum L.): a review, British Journal of Nutrition 108 (S1) (2012) S11-S26, doi: 10.1017/S0007114512000797.

[20] D. Rachwa-Rosiak, E. Nebesny, G. Budryn, Chickpeas—Composition, Nutritional Value, Health Benefits, Application to Bread and Snacks: A Review, Crit Rev Food Sci Nutr 55 (8) (2015) 1137-45, doi: 10.1080/10408398.2012.687418.

[21] R.K. Gupta, K. Gupta, A. Sharma, M. Das, I.A. Ansari, P.D. Dwivedi, Health Risks and Benefits of Chickpea (Cicer arietinum) Consumption, J Agric Food Chem 65 (1) (2017) 6-22, doi: 10.1021/acs.jafc.6b02629.

[22] A. Paço, C. Brígido, A. Alexandre, P.F. Mateos, S. Oliveira, The Symbiotic Performance of Chickpea Rhizobia Can Be Improved by Additional Copies of the *clpB* Chaperone Gene, PLOS ONE 11 (2) (2016) e0148221, doi: 10.1371/journal.pone.0148221.

[23] U.C. Jha, Current advances in chickpea genomics: applications and future perspectives, Plant Cell Rep 37 (7) (2018) 947-65, doi: 10.1007/s00299-018-2305-6.

[24] M. Kumar, M.A. Yusuf, M. Nigam, M. Kumar, An Update on Genetic Modification of Chickpea for Increased Yield and Stress Tolerance, Mol Biotechnol 60 (8) (2018) 651-63, doi: 10.1007/s12033-018-0096-1.

[25] J. Sheen, Signal Transduction in Maize and Arabidopsis Mesophyll Protoplasts, Plant Physiol 127 (4) (2001) 1466-75, doi: 10.1104/pp.010820.

[26] T. Eeckhaut, P.S. Lakshmanan, D. Deryckere, E. Van Bockstaele, J. Van Huylenbroeck, Progress in plant protoplast research, Planta 238 (6) (2013) 991-1003, doi: 10.1007/s00425-013-1936-7.

[27] V. Marx, Plants: a tool box of cell-based assays, Nat Methods 13 (2016) 551, doi: 10.1038/nmeth.3900.

[28] Q. Shan, Y. Wang, J. Li, C. Gao, Genome editing in rice and wheat using the CRISPR/Cas system, Nature Protocols 9 (2014) 2395, doi: 10.1038/nprot.2014.157.

[29] T. Čermák, N.J. Baltes, R. Čegan, Y. Zhang, D.F. Voytas, High-frequency, precise modification of the tomato genome, Genome Biology 16 (1) (2015) 232, doi: 10.1186/s13059-015-0796-9.

[30] G. Liang, H. Zhang, D. Lou, D. Yu, Selection of highly efficient sgRNAs for CRISPR/Cas9-based plant genome editing, Scientific Reports 6 (1) (2016) 21451, doi: 10.1038/srep21451.

[31] C.-S. Lin, C.-T. Hsu, L.-H. Yang, L.-Y. Lee, J.-Y. Fu, Q.-W. Cheng, F.-H. Wu, H.C.-W. Hsiao, Y. Zhang, R. Zhang, W.-J. Chang, C.-T. Yu, W. Wang, L.-J. Liao, S.B. Gelvin, M.-C. Shih, Application of protoplast technology to CRISPR/Cas9 mutagenesis: from single-cell mutation detection to mutant plant regeneration, Plant Biotechnol J 16 (7) (2018) 1295-310, doi: doi:10.1111/pbi.12870.

[32] J.W. Woo, J. Kim, S.I. Kwon, C. Corvalán, S.W. Cho, H. Kim, S.-G. Kim, S.-T. Kim, S. Choe, J.-S. Kim, DNA-free genome editing in plants with preassembled CRISPR-Cas9 ribonucleoproteins, Nat Biotechnol 33 (2015) 1162, doi: 10.1038/nbt.3389.

[33] Y. Zhang, Z. Liang, Y. Zong, Y. Wang, J. Liu, K. Chen, J.-L. Qiu, C. Gao, Efficient and transgene-free genome editing in wheat through transient expression of CRISPR/Cas9 DNA or RNA, Nature Communications 7 (1) (2016) 12617, doi: 10.1038/ncomms12617.

[34] H. Kim, S.-T. Kim, J. Ryu, B.-C. Kang, J.-S. Kim, S.-G. Kim, CRISPR/Cpf1-mediated DNA-free plant genome editing, Nature Communications 8 (2017) 14406, doi: 10.1038/ncomms14406.

[35] M. Malnoy, R. Viola, M.-H. Jung, O.-J. Koo, S. Kim, J.-S. Kim, R. Velasco, C. Nagamangala Kanchiswamy, DNA-Free Genetically Edited Grapevine and Apple Protoplast Using CRISPR/Cas9 Ribonucleoproteins, Frontiers in Plant Science 7 (1904) (2016), doi: 10.3389/fpls.2016.01904.

[36] Li J.-F., Chung H.S., Niu Y., Bush J., McCormack M., Sheen J., Comprehensive protein-based artificial microRNA screens for effective gene silencing in plants Plant Cell 25 (5) (2013) 1507-1522 , doi: 10.1105/tpc.113.112235.

**Supplementary materials**

**
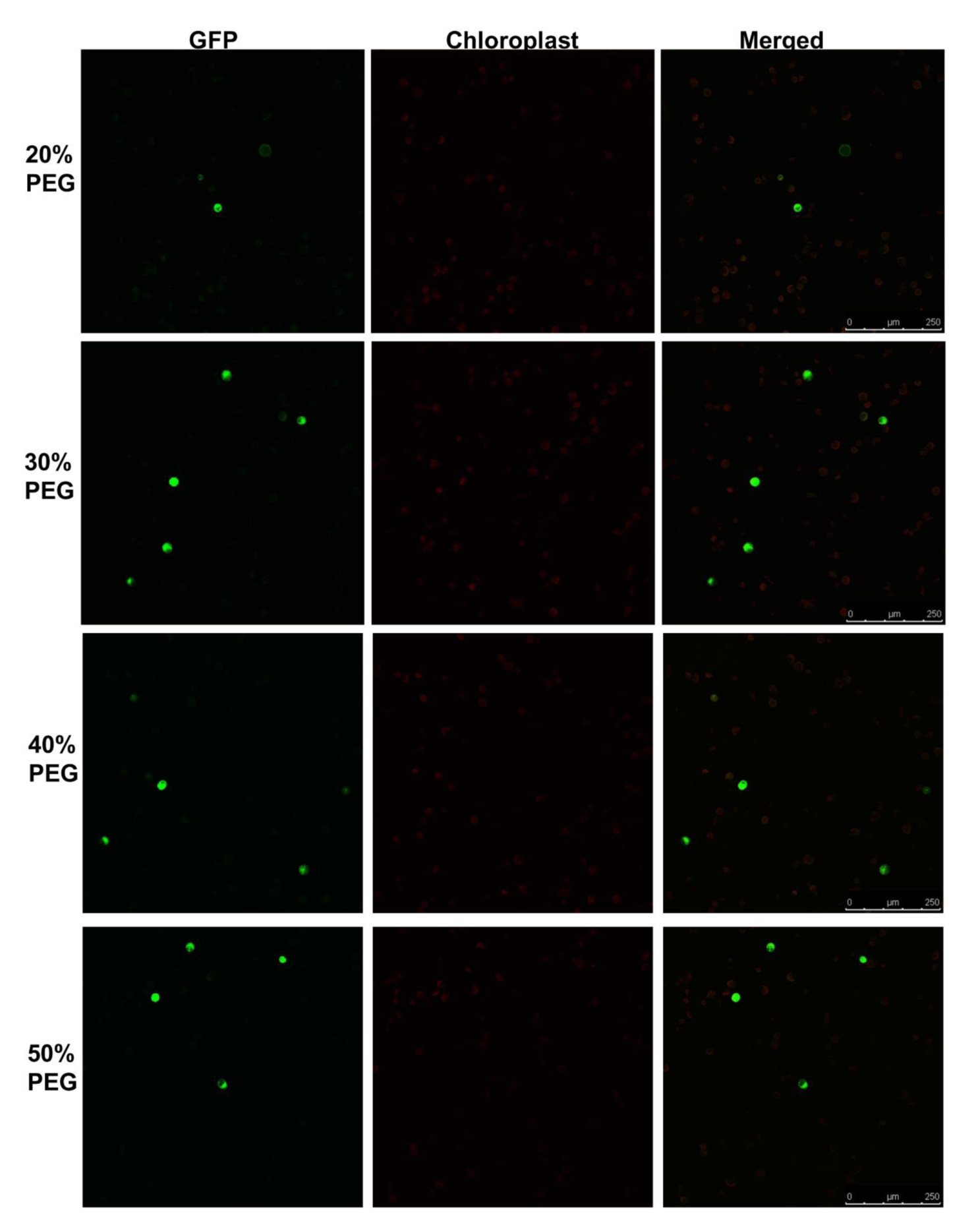
**

**S1 Fig. Chickpea mesophyll protoplast transfection efficiency.** Confocal images showing transfection efficiency of chickpea mesophyll protoplasts transformed with 5 µg plasmid DNA and with different initial concentrations of PEG. Green and chloroplast fluorescence signals were merged. Fluorescent signals of GFP fusion proteins were imaged 16hr after transfection. Scale bars=250µm. (TIF)


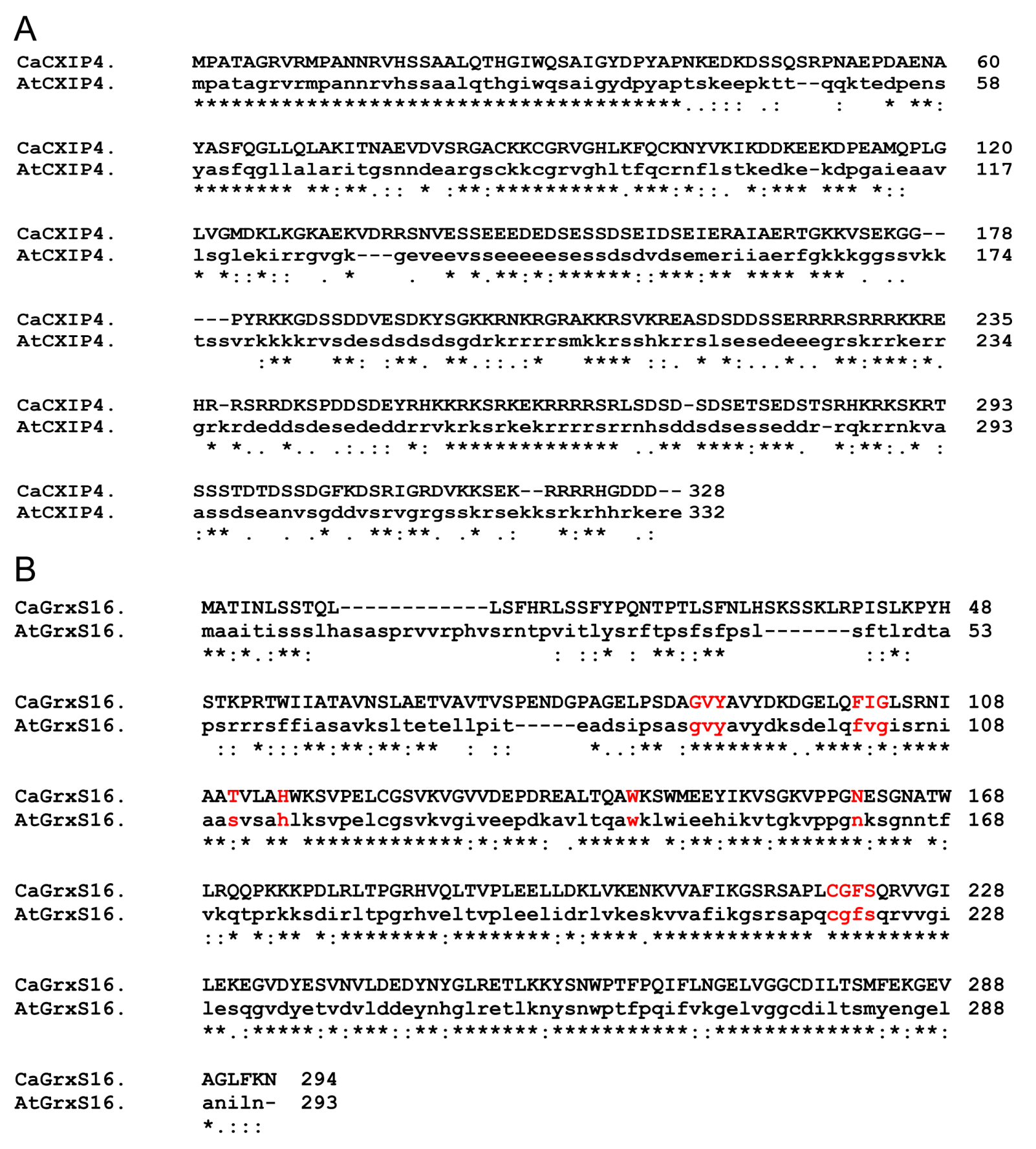


**S2 Fig. Amino acid sequence alignment of Chickpea** **with Arabidopsis homologs**. Alignments of Chickpea CXIP4 (A) and GrxS16 (B) with Arabidopsis homologs were performed using the Clustal Omega program. In (B), highlights indicate the GIY-YIG endonuclease domain and its key amino acid residues, and the Grx CGFS motif. (TIF)
